# What is Normal? The Impact of Psychiatric Classification on Mental Health Practice and Research

**DOI:** 10.3389/fpubh.2013.00068

**Published:** 2013-12-09

**Authors:** Wulf Rössler

**Affiliations:** ^1^Collegium Helveticum, University of Zurich and Eidgenössische Technische Hochschule Zürich (ETHZ), Zurich, Switzerland; ^2^University of Lüneburg, Lüneburg, Germany; ^3^Laboratory of Neuroscience (LIM 27), Institute of Psychiatry, University of Sao Paulo, Sao Paulo, Brazil

**Keywords:** psychiatric classification, case identification, DSM-5, psychiatric epidemiology, subthreshold disorder

Psychiatry is a rather quite young medical discipline, psychiatry evolved into its present form at the beginning of the nineteenth century (1). From its beginning there has been a discussion about the classification of mental disorders. The modern classification systems originated during the middle of the last century. In 1949 a section on mental disorders was added to the International Classification of Diseases (ICD) of the World Health Organization (WHO). The first Diagnostic and Statistical Manual (DSM) of the American Psychiatric Association was published in 1952 and listed 106 mental disorders (2).

The 1960s saw persistent attacks on the field of psychiatry from so-called anti-psychiatrists [e.g., (3)]. During that time these opponents assumed that the main purpose of psychiatric classification was to discipline maladjusted individuals. This “Zeitgeist” was best expressed in the movie “One Flew over the Cuckoo’s Nest,” which portrayed a repressive psychiatric system intent on enforcing “normal” behavior through electroshocks.

Despite all of the social criticism, psychiatric professionals continued to elaborate on the original classification system, resulting in a second and third version of the DSM. These revisions were accompanied by a continuous increase in the number of mental disorders. For example, DSM-III contained 265 diagnostic categories while DSM-IV, introduced in 1994, listed 297 psychiatric disorders. Accordingly, revisions of the ICD were published.

In 2013, the most recent edition, DSM-5, was released. Prior to this, fierce debate about the conceptual issues of psychiatric classification spilled over into the public media worldwide. Previous revisions that had followed the period of antipsychiatry [including those by ICD, a competing classification system of the WHO] mostly went unchallenged or were discussed only within academic circles of experts.

Although current criticism has been widespread, one person in particular has given a face to the voice of those critics. Allen Francis, Professor Emeritus of Psychiatry at Duke University, is the former chairman of the taskforce that revised DSM-III, resulting in DSM-IV. Even if his critique was very much influenced by the predicted impact of those revisions on the U.S. mental health care system, he turned the knife in a wound of psychiatry, i.e., challenging the concept of psychiatric classification. Notably, he argued that lowering the threshold for psychiatric diagnoses would lead to an undue increase in the number of persons labeled in such a way, with corresponding consequences not only to them but also to the health care system itself (4).

## What are the Consequences of Psychiatric Classification?

The introduction of an agreed psychiatric classification system has been a milestone because it has enabled researchers and psychiatric professionals worldwide to communicate about diagnoses in a standardized manner. Prior to this, these professionals had used the same words, but with widely varying concepts and content. This was demonstrated by Wing (5), who compared rates of schizophrenia between the United Kingdom and the United States. This critical situation resulted in a demand for a standardized psychiatric classification (6).

In many respects – for better or for worse – psychiatric diagnoses have a direct impact on the lives of the persons concerned and on the health care systems that provide assistance. For example, one of the more positive aspects of receiving a psychiatric diagnosis is that a person can then take advantage of all of the social and medical benefits associated with such an illness. Medical care and psychiatric treatment, inevitably requires a respective medical/psychiatric diagnosis. Further important purposes are, that medical services are reimbursed by health insurance providers only on the basis of (psychiatric) diagnoses. Likewise, licensing authorities, e.g., for pharmacological treatment, make their decisions based on those diagnoses.

Within the industrial world, the growing importance of psychiatric diagnoses might be exemplified by the fact that approximately 20% of the working-age population now suffers from diagnosed mental disorders (7). Public health statistics from Germany showed that the number of persons on sick leave because of these disorders nearly doubled from 1994 to 2010. In that final survey year, such disorders accounted for 9.1% of all sick-leave absences, averaging 23.4 days per person per annum (8). The same is true for pension funds. Mental disorders, notably depression, are a leading cause of employment disability. The need for premature disability benefits because of mental illness has placed a great burden on pension funds in Europe. In 2001, approximately 33.3% of all persons receiving such benefits in Switzerland had a psychiatric diagnosis; by 2010, this proportion had increased to 41.9% (9). Finally, there is an important interface of psychiatric diagnoses with the legal system. Courts make forensic decisions based on such diagnoses. Currently, the best-known forensic case worldwide is probably that of the Norwegian mass murderer Anders Breivik, who killed 77 people in a massacre in 2011. Did he suffer from a psychotic disorder or not? This was the decisive question for the conviction following his court hearing, namely the question of whether he was accountable for this crime (10).

For the affected person, one of the negative sides of receiving a psychiatric diagnosis is that he or she can become labeled and stigmatized as being mentally ill. This stigma has been extensively researched. The universality of mental illness and the accompanying stigma are widely acknowledged across cultures, including countries in the Western Hemisphere (11), Asia (12), and Latin America (13). Prior to being given a psychiatric diagnosis, one might be regarded as simply an odd or eccentric person. Once diagnosed, however, negative labels are then applied, such as that persons with mental illness are dangerous and aggressive. In turn, this stereotype enhances the social distance to those people (14). The stigma experienced from family members is also omnipresent (15, 16). This societal rejection adds to the barriers encountered when seeking help and contributes to this stigmatization. Mental health professionals often might also have negative attitudes toward persons with these illnesses (17, 18). Furthermore, the shame that results from being in an environment fraught with these rejections is a kind of self-stigmatization that presents another barrier to appropriate help-seeking (19).

## Some Flaws in Psychiatric Classification

Aside from the obvious issues directly related to affected persons and their caregivers, there are several theoretical considerations concerning psychiatric classification. Diagnoses should tell us something about the etiology and pathogenesis of an illness. However, both DSM and ICD claim to be a-theoretical. Their standards refer primarily to the reliability, especially test–retest reliability, of the applied categories. The fact that training within the standard classification systems that are currently being used produces acceptable inter-rater consistency is not very informative about the validity of psychiatric diagnoses. Furthermore, little attention has been paid to the fact that one’s understanding of mental symptoms is embedded in a network of cultural meanings that vary from one nation to another, or even among different groups within a single nation (20).

Psychiatric diagnoses are, firstly, conceptualized along the medical illness model. Medical diagnoses arise from symptoms or signs that indicate an underlying somatic disorder. By contrast, psychiatric researchers have not yet been able to find a biological substrate or laboratory marker for identifying mental disorders. Instead, biological studies within the field of psychiatry have presented only overwhelming evidence for mean differences between persons with a certain diagnosis and so-called healthy controls (N.B., a “healthy” person is generally defined as one who is not utilizing a mental health care system). With regard to biological markers, however, a strong overlap exists between mentally ill persons under investigation and healthy controls, making it impossible to separate the ill from the healthy. Thus, all psychiatric diagnoses to-date have relied exclusively upon clinical assessments.

## Some Disturbing Results from Epidemiological Surveys

During the last decades, highly structured research interviews have been developed that allow for a reliable assessment of mental symptoms and, concurrently, the identification of “cases” within large population samples. Unfortunately, due to differences in the construction of these instruments, some of the best-known general-population surveys have produced quite different rates for individual disorders. For example, Bijl et al. (21) analyzed prevalence rates from Canada, Chile, Germany, The Netherlands, and the United States. Estimates for 12-month prevalence, i.e., the proportion of a population under investigation that has experienced a mental disorder during the past year, ranged between 17.0% for Chile and 29.1% for the United States (Canada 19.9%, Germany 22.8%, The Netherlands 24.4%). Regier et al. (22) compared two large-scale surveys conducted in North America at approximately the same time – the “Epidemiological Catchment Area Study” (ECA) and the “National Comorbidity Survey” (NCS). Using the former, they calculated selected prevalence rates of 4.1, 4.2, 9.9, 1.1, and 1.6% for diagnoses of alcohol dependency, major depression, anxiety disorder, panic disorder, and social phobia, respectively, versus 7.4, 10.1, 15.3, 2.2, and 7.4% when the latter survey was applied. Similar differences were found when lifetime prevalence rates were compared.

It is difficult to interpret such divergent figures, and to identify the magnitude of the population in need, if we do not assume that those differences in values are likely due to difficulties in making reliable case assessments (23). Not only do these contrasting data raise concerns about the comparability of different studies but assessments that reveal consistently high rates also invite serious questions about the clinical significance of all of these disorders. Thus far, the discussion within the psychiatric community has made clear that symptoms are not sufficient when defining a group of persons in need (24). One major change to DSM-IV was the inclusion of a criterion for clinical significance, which required that symptoms cause “clinically significant distress or impairment in social, occupational, or other important areas of functioning.” Thus, this rule attempted to minimize false-positive diagnoses in situations where the symptom criteria did not necessarily indicate pathology.

## A New Approach to Making a Psychiatric Classification

Currently, categorical classification systems constitute agreed-upon definitions for pragmatically assigning mental illnesses. Most mental states are generally thought of as dichotomous entities that can be identified by applying certain operationalized criteria. In a clinical mental health setting one is naturally inclined to think that these states obviously are “cases” that need treatment. Those cases have been deduced from clinical observations of severely ill individuals who have passed through various filters of help-seeking. This clinical perspective, however, cannot be taken as evidence that these conditions exist as such in nature; instead, they represent the endpoints of a continuous characteristic (25). However, psychiatric diagnoses do not constitute natural illness entities. They are categories without natural boundaries. Because most human behavior is located along a continuum, no clear cut-off point exists to separate good health from illness and, as such, to define a point where the need for treatment exactly starts. Contrary to the situation in clinical practice, disease at the general-population level exists overall as a continuum rather than as an all-or-none phenomenon. This is also true for physiological processes. Thus, blood pressure and glucose tolerance are continuously distributed characteristics within the general-population. However, because the clinical decision to treat is dichotomous, terms like “hypertension” and “diabetes” are used in medicine.

This continuum approach has been widely accepted when dealing with affective disorders (26). Debate is now emerging about whether one can also take this approach in conjunction with psychotic disorders (25). Within a general-population, van Os et al. (27) have calculated a rate of approximately 5% for psychotic symptoms that are below the threshold of a psychotic disorder. That percentage is five times higher than that reported for full-blown schizophrenia. Using data gathered for over 30 years from a community cohort in the Canton of Zurich, we have realized that those symptoms are associated with significant dysfunctions in social roles (28). As such, those symptoms are of clinical importance because their presence can also increase the risk for co-morbid mental disorders (29), including those related to substance use (30). Hence, with regard to schizophrenia-spectrum disorders, the reference population is obviously much larger than what has been commonly assumed. Because persons affected at a threshold below that of a psychosis diagnosis are subjectively distressed and in need of help, it is quite likely that physicians “upgrade” the symptomatology to a suitable psychiatric diagnosis, which then officially allows them to pursue psychiatric treatment. From the professional perspective toward sub-clinical disorders, a significant proportion of persons with sub-clinical psychosis displays mental symptoms that are, to a varying degree, accompanied by functional disability.

In a next step, it then becomes essential to understand what actually causes an individual at some position along the continuum to become a clinical “case.” An investigation of the psychological and environmental mechanisms that result in psychotic symptoms would be particularly important in view of current interest in preventing individuals from transitioning from non-clinical to clinical psychotic states. That is, the goal would be to keep a person with psychotic symptoms from becoming a patient with a psychotic disorder. Although there may be a continuous relationship between symptoms and disorder without major discontinuity, it is possible that psychotic symptoms behave like hypertension, which is on a direct continuum with constant, normal fluctuations in blood pressure. Although not symptomatic in itself, beyond a certain threshold the risk of somatic complications involving other organs increases exponentially for a hypertensive individual.

It is quite obvious that psychiatric classification is not only a matter of academic debate but also has a direct impact on the lives of the affected and their families. However, if we continue to cling to the current categorical approach, we will never be able to abandon this discussion and will instead create an inflation of mental illness by lowering the threshold of psychiatric diagnoses or, because of restrictive diagnostic criteria, miss those who are truly mentally ill.

By applying a continuum approach many of these problems could be resolved, even if this does not meet the approval of health insurance and other social security stakeholders. This procedure would allow professionals to administer flexible treatments wherever there is significant personal distress and/or significant functional disability concerning social roles. This would then address predominantly the interests of psychiatric patients. In doing so, we would not need to discuss whether we are creating diagnostic inflation or false epidemics, per Allen Francis. Instead, it would be left to personal dialogs between concerned individuals and qualified personnel to decide when and where professional help and support are necessary. This also would be advantageous to the psychiatric profession because in no other medical discipline do patients express more hesitation about receiving assistance. Finally, a continuum approach would be beneficial for psychiatric researchers because the categorical, traditional approach has meant the loss of important data about all sub-threshold disorders. Including such previously missing information would provide us with new insights into the onset and course of mental disorders [e.g., (31)].
